# The “Toronto prosthesis”, an appealing method for restoring patients candidates for hybrid overdentures: A case report

**DOI:** 10.4317/jced.50877

**Published:** 2012-12-01

**Authors:** Javier Montero, Carla Macedo de Paula, Alberto Albaladejo

**Affiliations:** 1 DDS. Graduate in Odontology. PhD in Dentistry Tenured Lecturer of Prosthodontics. Faculty of Medicine. University of Salamanca. Campus Miguel de Unamuno. Salamanca; 2 DDS. Graduate in Odontology. Postgraduate Dental Student. Faculty of Medicine. University of Salamanca. Campus Miguel de Unamuno. Salamanca; 3 DDS. Graduate in Odontology. PhD in Dentistry Tenured Lecturer of Orthodontics. Faculty of Medicine. University of Salamanca. Campus Miguel de Unamuno. Salamanca

## Abstract

The implant is a therapeutic resource in constant evolution, and the different types of implants and techniques have been increasingly used in cases of both fully or partially edentulous patients. In some cases they provide more conservative treatment, and in others better stability, retention, and function. To achieve a satisfactory result, there are several factors that should be taken into account: the type and quality of the bone, bone density, the placement location of implants, retrievability of restorations, the patient’s motivation, and economic issues. Trainees should be aware of the limitations of the techniques that can be used for successful prosthetic rehabilitation. This work describes the prosthetic rehabilitation of a fully edentulous mandible treated with dental implants using the ‘Toronto Bridge” technique for restoring both function and aesthetics. This type of prosthesis is a screwed-in mesostructure with milled abutments for the cementation of single or multiple suprastructures. This device could also be named “abutment-hybrid overdenture” The main advantages and disadvantages of this protocol are discussed.

** Key words:**Implant-supported restorations, dental implants.

## Introduction

Implant therapy has evolved rapidly and indeed highly satisfactorily in recent years. The desire to achieve predictable results has in the long-run involved several issues concerning the materials, techniques and anchorages used. Regarding the types of connection between the implant and restoration, these can be screwed, cemented, or a technique combining both can be implemented. Comparative analyses of both screw-retained and cement-retained prostheses have been published elsewhere (1-3), the authors concluding that the best advantage of the former is retrievability while the latter provides a good passive fit between structures (implant-abutment interfaces) and better aesthetics (mimetism). Nevertheless, there is still a lack of a gold standard for the type of anchorage to be used.

Traditionally, all implant-supported prostheses have been screwed in owing to the possibility of later removing and treating the implants. However, since 1988 Lewis et al. (4) developped the concept of cemented implant restorations. This trend began to overcome the aesthetic problems in tilted implants by mean the customization of a castable UCLA abutment (4).

The challenge to combine the advantages of screw- and cement-retained prostheses has led to the development of new prosthodontic techniques, such as the so-called Toronto Bridge, which is a screwed-in mesostructure with milled abutments for the cementation of single or multiple suprastructures.

Here we report the results of the prosthetic restoration of one totally edentulous patient using the “Toronto Bridge” approach.

## Case Report

A 63-year-old man with mandibular prognathism (classified as Angle Class III), attended a private dental office seeking full prosthetic rehabilitation. He had already be wearer of partial dentures in both arches and he wanted fixed prostheses. The maxilla was rehabilitated using both tooth and implant supported fixed partial dentures. For the lower jaw, after the clinical and radiological (Axial Tomography) assessment, it was decided that he was able to receive implant-supported rehabilitation, despite the insufficient bone height and width. We were aware that the mandibular restoration had to restore both the hard and soft tissues. An overdenture had been suggested previously, but the patient refused it. Thus, 7 externally hexed implants (MG Osseous. Mozograu SL, Valladolid, Spain) were inserted according to manufacturer recommendations at the level of 46/44/43/32/33/34/36 We used a single-phase technique: i.e., maintaining the healing abutments with a provisional relining material beneath the removable prosthesis, which was supported by 2 transitional natural teeth -31 and 41- during the period of implant osseointegration.

All implants healed uneventfully, but a surgical bur was broken during drilling due to the bone strength at the level of 47. Moreover it was found that the patient had an unknown blood disorder, detected in the surgical procedure and in the immediate postoperative phase. Later analysis confirmed the presence of haemophilia, although this finding had been no obstacle to the performance of the surgery.

**Figure 1:**
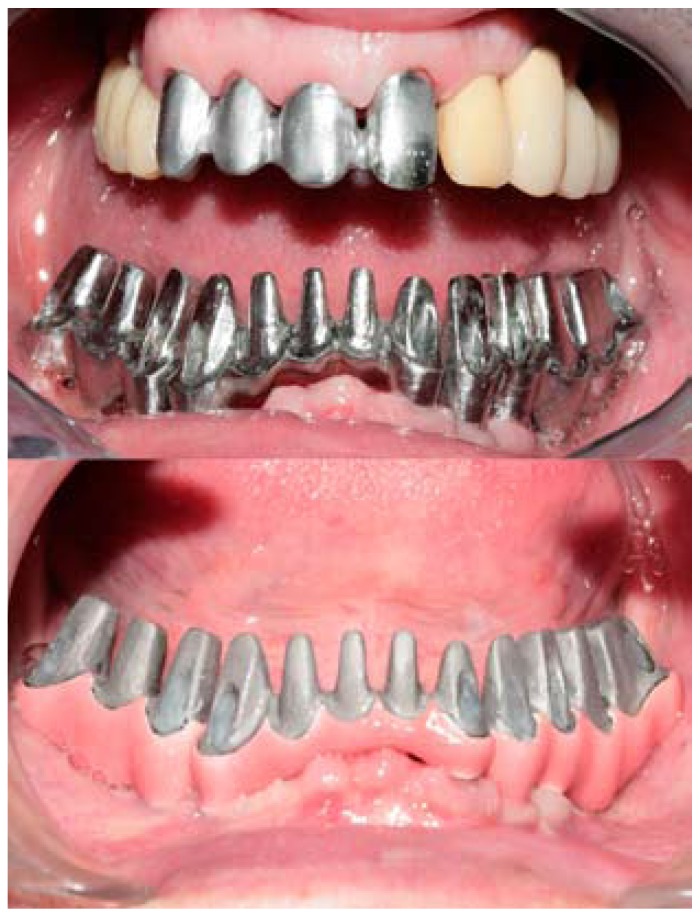
Frontal view of the sectioned mesostructure with multiple individual abutments before and after the ceramization in pink to mimic the soft tissues.

**Figure 2:**
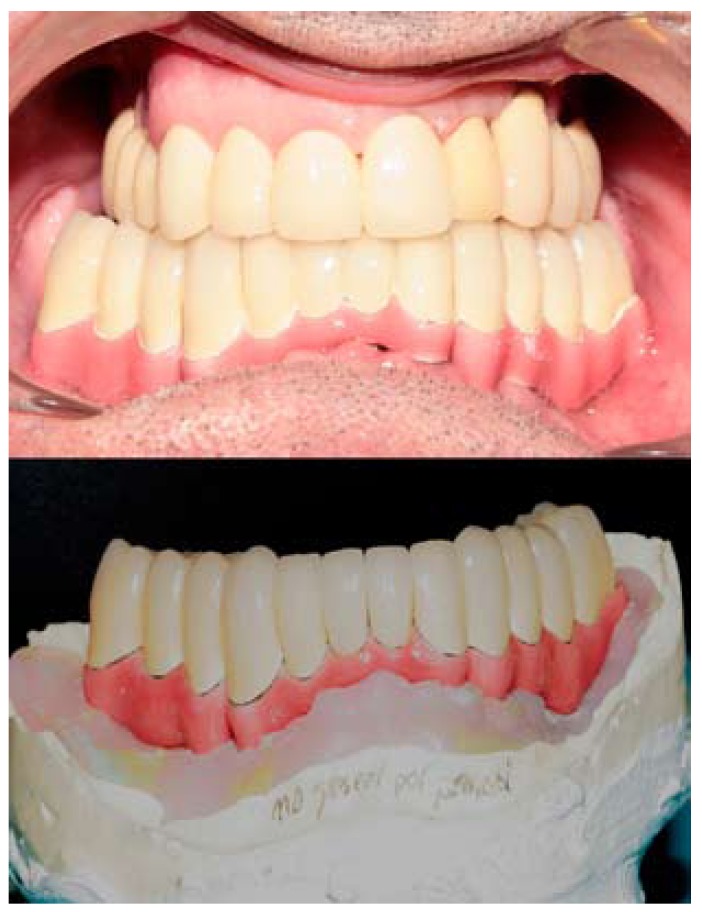
Porcelain fused to metal individual crowns placed on the mesostructure in the dental working cast and after the provisional cementation in mouth.

After 3 months, impressions were taken using the pickup technique and a test for occlusion and aesthetic composition was performed on a wax-up, to simulate the final restoration. Then, a sectioned mesostructure with multiple individual abutments was made by casting, and after a clinically acceptable passive fit had been con-firmed multiple individual crowns of metal-ceramic were made (Fig. 1). The mesostructure was ceramicized in pink to mimic the soft tissues (Fig. 1) and the crowns were provisionally cemented after the glazed ceramic crowns had been inserted (Fig. 2).

**Figure 3:**
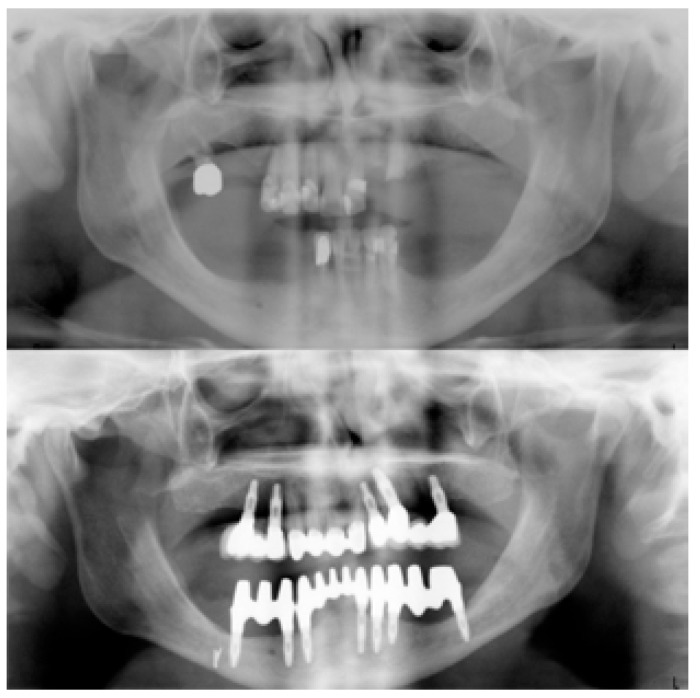
Preoperative and postoperative orthopantomographies: The maxilla was rehabilitated using both tooth and implant supported fixed partial dentures; and the lower jaw using the Toronto Prosthesis.

The “Toronto prosthesis” comprised 12 teeth made of porcelain fused to metal, supported by 7 implants distributed along the jaw in three sections (Fig. 3), so that the occlusal forces would not compromise the implants. The crowns were individual, with points of contacts but leaving empty spaces for proper hygiene. After rehabilitation, the patient improved his mandibular prognathism through functional mandibular retrusion, thus gaining a better aesthetic aspect. The end result provided an adequate contour to facilitate maintenance and healthy gum tissue.

## Discussion

Currently, the diversity of resources available in dentistry have led the patients to become more demanding, and with this, to seek more functional and more aesthetic restorations. In implant dentistry it is important that professionals should understand the effects of the mechanism of anchorage when choosing the type of retention of the prosthesis: screwed or cemented. The factors influencing the different anchoring methods are: 1) Ease of manufacture and cost; 2) the passivity of the fit; 3) Retention; 4) Occlusion; 5) Aesthetics; 6) prosthesis retrievability, and 7) implant ubication (1-3). However, the challenge of combining the advantages of both types of attachment encouraged authors to combined screw- and cement-retained prosthesis in implant prosthodontics (5), as the method report in this case.

In this case, the prosthesis is made of porcelain fused to metal, a material that has good characteristics in the sense of aesthetics, polish, a wide range of colour and longevity (6), being, to date, the best choice for recon-struction on implants (7). All the crowns were cemented provisionally on the individual abutments emerging from the mesostructure, allowing -depending on the area of the contact points- easy retrievability in the event of any repairs being necessary. The disadvantage is related to the time consuming nature of the technical procedure (adjusting contact point individually), which therefore is more complex and expensive for technicians.

One of the most important factors for the success of any implant-supported prosthesis is directly related to the precision of fit between the components that form the implant abutment (8,9). To achieve absolute passive fit, in a full arch superstructure screwed directly to implants, is not possible since the emergence of each implant hampers the insertion of a structure in a single axis (10). Therefore we sectioned the mesoestructure in 3 pieces in which implants had congruent axes. But also we could use transmucosal abutment with conical connections for compensating the implant angulations and allowing the proper settlement of a single structure on the transmucosal abutments. Both mode of construction will have possibility of removal but the former reduces costs and simplify the mode of reconstruction. In any case, the clinical fit-evaluation methods often do not detect “hidden” inaccuracies, so we assumed that certain misfit seems to be well tolerated by the implant-prosthesis system. The conventional and clinical procedures applied commonly seem able to afford a biological acceptable fit.

The origin of the term “Toronto Prosthesis” came from an extrapolation of the clinical and laboratory procedures presented by Professor George Zarb (recently retired as Professor and Head of Prosthodontics at the Faculty of Dentistry, University of Toronto) in the early 80s to a not indexed Italian dental journal. As a result, the methodology employed for designing the framework for fixed full arch prostheses due to the departmental research work at the University of Toronto was referred to as the “Toronto Bridge” by the European colleagues in their meeting presentations. However as the Toronto Group led by George Zarb has widened the implant prosthodontics methodologies, some other approaches have latter christened as Toronto Bridges or Toronto Prosthesis although they are distinct approaches of that report here (11). Thus since this denomination is today a confusing term, a possibly better descriptor would be an “abutment-hybrid overdenture”.

The main advantage of the Toronto Bridge is that it allows the dentist to correct implant emergence and that the milled abutment is sufficiently tapered to ensure retention of the crown by using provisional cement (retrievability). However the laboratory costs are higher than the conventional rehabilitation using either an acrylic hybrid overdenture or multiple implant-supported bridges screwed or cemented on transmucosal abutments.

## References

[1] Michalakis KX, Hirayama H, Garefis PD (2003). Cement-retained versus screw-retained implant restorations: a critical review. Int J Oral Maxillofac Implants.

[2] Lee A, Okayasu K, Wang HL (2010). Screw- versus cement-retained implant restorations: current concepts. Implant Dent.

[3] Chee W, Jivraj S (2006). Screw versus cemented implant supported restorations. Br Dent J.

[4] Lewis S, Beumer J, Hornburg W, Moy P (1988). The "UCLA" abutment. Int J Oral Maxillofac Implants.

[5] Preiskel HW, Tsolka P (2004). Cement - and screw-retained implant-supported prostheses: Up to 10 years of follow-up of a new design. Int J Oral Maxillofac Implants.

[6] Zarone F, Russo S, Sorrentino R (2011). From porcelain-fused-to-metal to zirconia: clinical and experimental considerations. Dent Mater.

[7] Heintze SD, Rousson V (2010). Survival of zirconia- and metal-supported fixed dental prostheses: a systematic review. Int J Prosthodont.

[8] Guichet DL, Caputto A, Choi H, Sorensen JA (2000). Passivity of fit and marginal opening in screw or cement-retained implant fixed partial denture designs. Int J Oral Maxillofac Implants.

[9] Taylor TD, Agar JR, Vogiatzi T (2000). Implant prosthodontics: Current perspective and future directions. Int J Oral Maxillofac Implants.

[10] Karl M, Winter W, Taylor TD, Heckmann SM (2004). In vitro study on passive fit in implant-supported 5-unit fixed partial dentures. Int J Oral Maxillofac Implants.

[11] Cicciù M, Risitano G, Maiorana C, Franceschini G (2009). Parametric analysis of the strength in the ''Toronto'' osseous-prosthesis system. Minerva Stomatol.

